# A Quorum-Sensing Factor in Vegetative *Dictyostelium Discoideum* Cells Revealed by Quantitative Migration Analysis

**DOI:** 10.1371/journal.pone.0026901

**Published:** 2011-11-03

**Authors:** Laurent Golé, Charlotte Rivière, Yoshinori Hayakawa, Jean-Paul Rieu

**Affiliations:** 1 Laboratoire de Physique de la Matière Condensée et Nanostructures, Université de Lyon, Université de Lyon I, CNRS, UMR 5586, Villeurbanne, France; 2 Center for Information Technology in Education, Tohoku University, Sendai, Japan; City of Hope National Medical Center and Beckman Research Institute, United States of America

## Abstract

**Background:**

Many cells communicate through the production of diffusible signaling molecules that accumulate and once a critical concentration has been reached, can activate or repress a number of target genes in a process termed quorum sensing (QS). In the social amoeba *Dictyostelium discoideum*, QS plays an important role during development. However little is known about its effect on cell migration especially in the growth phase.

**Methods and Findings:**

To investigate the role of cell density on cell migration in the growth phase, we use multisite timelapse microscopy and automated cell tracking. This analysis reveals a high heterogeneity within a given cell population, and the necessity to use large data sets to draw reliable conclusions on cell motion. In average, motion is persistent for short periods of time (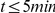), but normal diffusive behavior is recovered over longer time periods. The persistence times are positively correlated with the migrated distances. Interestingly, the migrated distance decreases as well with cell density. The adaptation of cell migration to cell density highlights the role of a secreted quorum sensing factor (QSF) on cell migration. Using a simple model describing the balance between the rate of QSF generation and the rate of QSF dilution, we were able to gather all experimental results into a single master curve, showing a sharp cell transition between high and low motile behaviors with increasing QSF.

**Conclusion:**

This study unambiguously demonstrates the central role played by QSF on amoeboid motion in the growth phase.

## Introduction

Cell migration is a central process in a number of normal and pathological situations including morphogenesis, immune system response and metastasis spreading. Directed cell migration in response to chemoattractants has been thoroughly investigated during past few years by different scientific communities (molecular and cellular biology [1], [2], physics and biophysics [3], [4]). More recently, the analysis of spontaneous cell movement in the absence of any directional stimuli has been the object of intense investigations that try to quantify and describe cell motion [5]–[14]. Cell movement has been classically described as a persistent random walk following the Ornstein-Uhlenbeck process [15]. This model derives from Langevin equation of motion, with white-noise. Accordingly, cells follow a directed motion over a short time range, while recovering normal Brownian diffusion over longer periods. The cross-over defines a persistence time [16]. More recently, many studies have pointed to the existence of anomalous behavior (*i.e.*, even at long time scale, the cells do not show Brownian motion) in mammalian cells [6], and amoebas [9]. To our knowledge, none of these biophysical studies [5]–[14] have investigated nor suggested the role of quorum sensing mechanisms in the regulation of spontaneous cell movement. It is however interesting to note that in the absence of external signals (very diluted conditions), Li *et al.* [7] have found a much faster migration than others using the same cell type but a higher cell density [9], [10].

Development in multicellular organisms requires quorum sensing (QS) mechanisms that allow monitoring of the density of different cell types. QS is accomplished by simultaneously secreting and sensing autocrine factors that accumulate in the extracellular space in a cell-density dependent manner. The concentration of these factors turns on signaling cascades which change the developmental program of the cell. QS has been described in detail for many bacterial systems [17], fungi [18], *Dictyostelium discoideum* [19] and was recently suggested to regulate ovarian cancer metastasis [20]. The simple *Dictyostelium* developmental cycle provides an excellent system in which to study eukaryotic QS. As long as nutrients are present, *Dictyostelium* cells multiply as unicellular amoeba (vegetative growth). However, when cells deplete their food source and begin to starve, they enter a developmental cycle and signal other cells by secreting an array of factors such as the glycoprotein conditioned medium factor (CMF) [21]. After more and more cells in a population have starved, the extracellular CMF reaches a threshold that activates CMF receptor, inihibiting PldB activity and thus increasing cAMP signaling [22]. This enables the cells to aggregate using relayed pulses of cyclic adenosine monophosphate(cAMP) as a chemoattractant. The expression of specific classes of genes is altered, the cells become polarized, move toward the source of cAMP and form streams that flow toward the aggregation center. This process forms a number of groups of up to 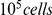. The group size is controlled by counting factors (CF) that mediate cell density sensing during the late aggregation and regulate myosin II distribution, motility and cell adhesion [23]. *Dictyostelium* cells also communicate during vegetative growth, although QS mechanisms are less understood. A recent analysis of the transcriptome of vegetative *Dictyostelium* at high cell density revealed not only the expression at moderate to low levels of countin (one of the components of CF) and CMF, but also the expression at low levels of rcdGG, another proposed quorum-sensing systems [24]. In addition, various types of prestarvation factors (PSFs) are continuously secreted during growth and accumulate in the medium in proportion to cell density [19], [25]. PSFs induce early developmental gene expression like discoidin in a dose-dependent manner and coordinate the initiation of multicellular development. AprA and CfaD are proteins secreted by vegetative cells that inhibit cell proliferation in a concentration dependent manner [26]. Recently a new quorum-sensing molecule (unfortunately not purified) was reported to regulate cell adhesion in vegetative *Dictyostelium* cells [27]. At high cellular densities, a strong decrease in cell adhesion and in the expression of the adhesion protein sibC was observed. The effect on cell motility of these various factors affecting gene expression has never been investigated.

It is well known that individual *Dictyostelium* cells exhibit variable motile properties and that even their average properties are often changing from one experiment to the other depending on unidentified parameters [23], [27]. It is therefore important to be able as much as possible to investigate the dependence of properties of cell motility on different experimental parameters in parallel. In this paper we investigate in a quantitative manner the role of cell density on the random motion of vegetative cells using a very large data set. We have characterized the motion with different parameters classically used for the analysis of motion of colloids in complex fluids or biological cells (mean square displacement, velocity autocorrelation, persistence time, bimodal analysis). Our results demonstrate the role of QS factors (QSF) in the spontaneous migration of amoeboid cells.

## Results

The role played by cell-density on spontaneous cell migration was analyzed thanks to statistical analysis of cell centroid displacement over time in different experimental conditions. First, cell density has been varied in a large range (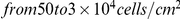, density experiments) corresponding to mean cell-cell distances ranging 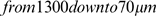). Second, in order to highlight the role of QSF secreted by cells in their spontaneous migration, the evolution of cell migration was analyzed over time (aging experiments), in conditioned media and under controlled flow conditions (flow experiments).

### A Random Motion With Persistence

**Figure 1:**
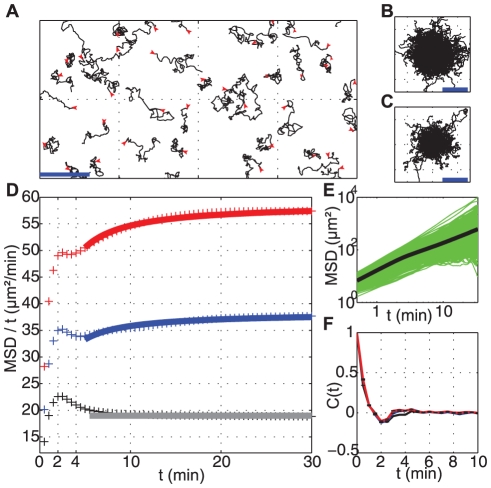
Cell track analysis. (**A**) Examples of cell trajectories lasting 50 min obtained at a cell density of 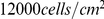. Red arrows represent starting points. (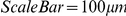); (**B**–**C**) Typical cell tracks, with their origins brought to a common point, are shown for (**B**) a condition of fast cell displacement (under a perfusion flow of 30 mL/h), and (**C**) a condition of slow cell displacement (same cell density without flow). (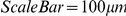); (**D**) 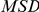 divided by time lag as a function of time lag for 3 typical cell conditions: red crosses, fast moving cells 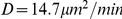; blue crosses, intermediate moving cells 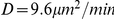; black crosses: slow moving cells 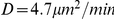. Solid lines are fits using the Fürth's formula. This plot reveals an overshoot at 2 min. The slower the cells, the larger the overshoot; (**E**) Mean Square Displacement (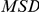) as a function of time lag 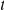 in log-log scale. Green lines: individual 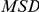 for each tracked cells, revealing the large dispersion of our population. Black bold line: Average 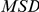 of all tracked cells; (**F**) Velocity autocorrelation C(t) as a function of time lag for the same cell conditions as (**D**), showing a negative peak at 2 min.

Fig. 1A displays the trajectories of about 30 cells tracked during 50 min with a sampling interval of 30 sec. The region corresponds to about one fourth of the full recorded frame. Using a motorized stage, we may typically record 20 different frames every 30 sec at different locations and in different wells. For this particular experimental condition (*i.e.*, cells submitted to a perfusion flow at 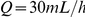), cell density and the number of tracked cells are high (*i.e.*, 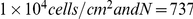, respectively). Trajectories look random (typical images and movies of cell migration using our spatio-temporal resolution are given in Supplementary material). Trajectories are displayed in Fig. 1B by overlapping their starting points. This graph shows the absence of any bias in the trajectories and the amplitude of the fastest cells. For another condition (*i.e.*, cells at similar density but without flow), cells are slower but still migrate randomly (Fig. 1C) (see supplementary Movie S2 for a comparison of these 2 conditions).

To quantify the randomness of these trajectories, it is useful to compute mean squared displacements (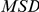). This can be done for each cell (represented as a green line in Fig. 1D) and a mean 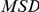 can be computed (thick black line in Fig. 1D). The first striking characteristic is the large dispersion of individual 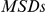 already noticed a long time ago in the pioneering work of Potel and MacKay [28].

The relative standard error (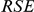) on the 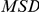 at 15 min is about 4.5% at 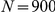, 13.5% at 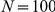 and increases dramatically if 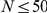 (see Supplementary Fig. S8A). A large number of cells is essential to get accurate 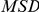 values. Statistics may be improved as well by increasing the total recording time (see Supplementary Fig. S8B). However, as we are interested in the long term behavior (where normal diffusion is recovered), there is no point to decrease the time interval between frames (here 30 sec) below the expected persistence time (see Supplementary Fig. S8B–C).

For each condition analyzed, the plot of the mean 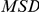 *vs.* time lag 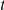 shows two apparent slopes in a log-log scale (Fig. 1E). It is linear at long times (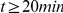) indicating normal (random) diffusion. At short times, it scales as 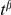 with an exponent 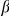 larger than one indicating a persistent motion. However data cannot be fitted over all times with the standard expression of random motion with persistence relation (Fürth's formula) [15]:
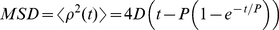
(1)


where 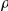 is the distance traveled, 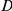 and 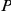 are the diffusion constant and persistent time, respectively. This is clearly evidenced by a 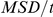 representation (Fig. 1D) which shows the presence of an initial overshoot near about 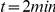. This overshoot has never been reported to our knowledge in previous studies but the reason could be that the 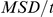 representation is not classically used. In log-log or linear 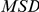 plots, we cannot notice this singularity.

The presence of this overshoot is correlated with the presence of a negative peak in the velocity autocorrelation function (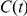) near 2 min (Fig. 1F). We have carefully checked that both are not due to noise on centroid positions (see Supplementary Fig. S3). The negative peak of 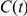 corresponds to an excess of turn angles larger than 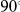 near 2 min: there is a significant probability that a cell moving in a given direction retracts backward after two minutes. It will be interesting to analyze pseudopod activity for those cells by using a larger spatial and temporal resolution but this is out of the scope of this work.

**Figure 2:**
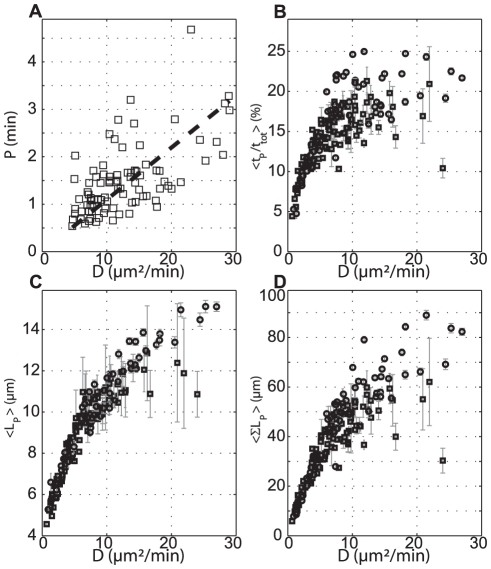
Cell persistence is correlated with Diffusion Coefficient *D.* All variables are plotted as a function of 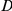 which is obtained (as well as the persistence time 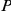) by fitting 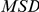 with the Fürth's formula at long times (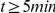). (**A**) Persistence time 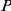 (values larger than the time interval of 30 sec were considered); (**B**–**C**–**D**) Parameters obtained from a bimodal analysis; (**B**) Ratio of time spent in persistent mode over total tracking time; (**C**) Length of persistence 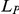 (distance end-to-end of each individual persistent portion of cell trajectory); (**D**) cumulative 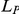, summed over all cell trajectory. Squares correspond to ‘Aging’ experiments, circles to Flow experiments. 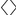 denotes averaging over all tracked cells in a given condition. Error bars represents SEM. The solid line in (**A**) is a linear fit 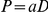 (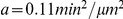, 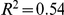).

For times greater than 2 min, the cell centroïd displacements show some slight persistence as velocity autocorrelation is significantly larger than zero especially for fast moving cells (red curve in Fig. 1F). For times much larger than the overshoot, it is possible to get a reasonable estimate of the persistence 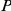 and of the diffusion constant 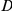 by fitting the mean 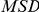 with the Fürth's formula. The reported values of 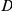 throughout the manuscript correspond to ones obtained by fitting data with a lower cut-off at 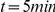. In average, one observes a positive correlation between 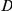 and 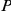: the larger the diffusion constant 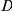, the larger is the persistence time, 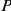 (Fig. 2A). The simplest relationship between these two parameters is a linear function. An intrinsic instantaneous speed 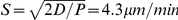 can be estimated from the slope of Fig. 2A which corresponds to the speed in the ballistic regime at short times in the Fürth's formula (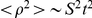 when 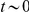). The existence of a constant intrinsic instantaneous speed would indicate that the long term migrated distance is caused by the ability of cells to maintain movement in a chosen direction, not by the increase in their intrinsic speed.

We also analyzed the statistics of the persistence portions of the trajectories with a bimodal analysis which was reported to be very helpful to describe the different kinds of motility from random to directed [29]. Fig. 2B–D show the relation between 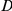 and the proportion of time spent in persistent mode 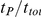, the mean persistent run length 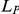 or the cumulated distance in the persistent mode 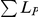, respectively. Experiments with faster cells have a larger mean 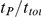 but also a larger 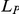. As a result the cumulated distance migrated in the persistence mode which is a combination of both 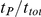 and 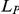 is very dependent on the conditions. Again, these parameters have a very broad distribution, accounting for the large cell-to-cell variability, but clear differences of the average values of each persistence parameters are found (Supplementary Fig. S5). This bimodal analysis confirms the strong correlation between persistence and diffusion constant. Even if the chosen resolution is low, it enables the analysis of the main modes of cell deformation. In supplementary Fig. S2 a correlation between cell shape and cell migration could be detected: the more cells are elongated the larger is the diffusion coefficient.

### Cells Move Faster at Low Cell Density

**Figure 3:**
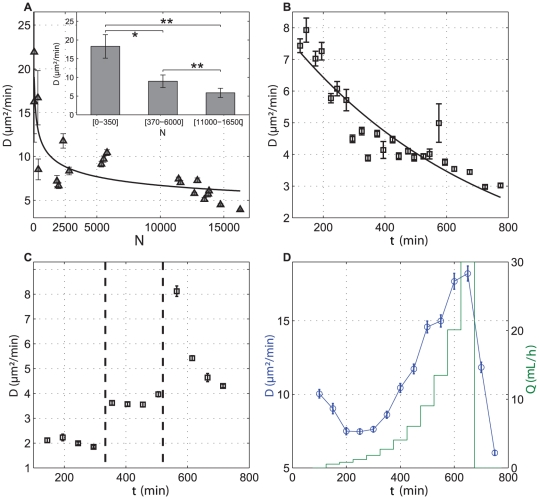
Cell motility depends on a QSF secreted by cells. The diffusion coefficient 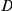 is decreasing with cell density and experimental time, highlighting the role played by a QSF factors in cell motion. Error bars represent SEM. (**A**) 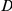 plotted as a function of the experimental cell density. Cells were recorded during 50 min, 1.5 hours after cells were washed in a fresh HL5 medium. Inset: Average 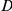 values and corresponding 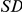 error bars obtained for different cell density ranges; (**B**) ‘Aging’ assay: as time increases QSF increases and 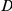 decreases; (**C**), Evolution of 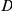 when medium is changed during experiment starting from Highly conditioned Medium (left part, during time [150–300 min], low 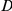), moderate conditioned medium (middle part, during time [350–500 min], intermediate 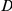) and fresh medium (right part, during time [550–700 min], rapid increase in 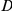, followed by an exponential decrease); (**D**) Evolution of 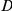 with flow 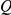. A home-made macrofluidic chamber enables renewing the flow. Applied flow is changed with exponential step (green line, vertical right axis). 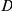 is first decreasing (corresponding to a state where QSF cell emission is overcoming QSF flow dilution), then increasing (corresponding to a state where QSF flow dilution is overcoming QSF cell emission). When flow is stopped after 700 min, 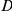 rapidly decreases (corresponding to a rapid increase in QSF concentration).

It is well known that starved cells acquire aggregation competence by exchanging signals with others at high cell density [30]. Aggregation-competent cells are highly polarized, persistent in their movement and move fast [31]. Surprisingly, vegetative cells behave in the opposite way: they move faster at low cell density. We checked this by measuring the mean 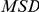 in a wide range of cell densities between 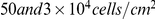 (Fig. 3A). The diffusion constant at the lower investigated density is five times larger than at the highest density. Differences are statistically significant (see inset of Fig. 3A).

### Cell Migration is Regulated by a Quorum Sensing Factor Secreted by Cells

Several tests were performed in order to check whether the dependence of cell migration on cell density could be regulated by an unknown factor secreted by cells.

First, we measured the evolution of the 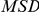 over time (“aging” experiments). For that, cell trajectories were continuously recorded during 12 hours and the 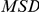 calculated during time intervals of 50 min. Time zero corresponds to the time when cells were washed and plated on the sample dish in fresh HL5 medium. The diffusion constant 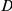 decreases from 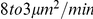 over 12 hours (Fig. 3B), whereas initial cell density is 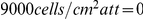. Clearly this change is not accounted for by the increase of the cell population (due to cell division) which is roughly only doubling during that experimental period, corresponding to a minor effect on 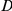 at a given time (Fig. 3A), and suggests the presence of a factor secreted by the cells over time.

Second, we studied the effect of various conditioned media (Fig. 3C). Highly conditioned medium (HCM) conditioned by growing cells in the exponential phase during 2 days strongly reduces 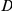 at 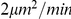 as compared to cells at the same density (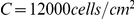) in fresh medium (Fig. 3A). Notice that 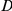 is very stable over 200 min. After that period of time, the medium of the same sample dish was exchanged for a moderately conditioned medium (MCM conditioned by cells at the same sample density, 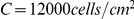 during 4 hours). This exchange induced an increase of 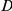 up to 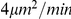 with little change during 200 min. This value is equal to the one obtained after the same period for aging experiments (Fig. 3B). Finally, the medium was once more exchanged to fresh HL5 and 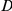 suddenly jumped to 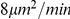 and slowly decreased with time with the same dynamics than in aging experiments (Fig. 3B). These experiments indicate that *(i)* the more conditioned the medium the slower the cell migration, *(ii)* migration rate saturates at very large conditioning and *(iii)* cells quickly reset their migrating properties as soon as the medium is modified (within the 50 min time interval necessary to record reliable 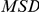 measurements).

Third, we perfused the sample dish with fresh medium with series of steps of increasing, albeit slow, flow rates (Fig. 3D). The duration of each step was 50 min and its amplitude roughly exponentially increasing up to 30 mL/hours where flow was stopped while cell trajectories were still recorded. With a high initial density (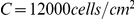) and for very slow flow rates (3 first steps), 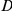 is first decreasing but afterwards it increases with increasing 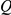 up to 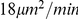. Once the flow is stopped, 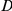 immediately decreases. It is known that shear stresses above 0.1 Pa trigger active actin cytoskeleton remodeling and shear-flow induced velocity along the flow direction [32]. In our experiments, the higher applied wall shear stresses (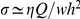 where 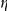, 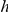 and 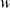 are medium viscosity, dish height and width, respectively) are much smaller, in the mPa range. We also checked that trajectories of perfused cell are isotropic (Fig. 1B) excluding therefore any shear-flow induced effect.

Complementary experiments with inhomogeneous samples show that local difference in cell density has a small effect on cell migration and tends to vanish with time (Supplementary Fig. S6). Local self organization of cells in territories was also investigated using pair correlation function 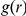, which quantifies the probability of finding other cells at distance 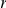 from each other (Supplementary Fig. S7). Results indicate that cell distribution is random, with no preferential organization over time. Finally, we checked that the diffusion constant value is independent of cell density in HCM. Very diluted cells (not shown) move with the same very low diffusion constant than cells at higher density (Fig. 3C) indicating that local cell-cell distance does not trigger the migration of cells.

All these experiments unambiguously demonstrate that cells are sensitive to an unknown quorum sensing factor (QSF) secreted by cells. This QSF accumulates in the medium (aging experiments), can be partially removed by an external flow (dilution) or can be concentrated up to level saturating cells (highly conditioned medium).

### A Simple Kinetics Model to Describe the QSF Concentration for each Experimental Situation

With simple assumptions, it is possible to model the ratio of the number 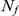 of secreted QSF over some synthesis rate 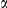. The first assumption is that 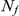 quickly reaches an equilibrium in the full sample volume. It holds if diffusion and or convection due to the motorized stage motion or the external perfusion if any are quickly equilibrating the density with respect to the typical 50 min experimental time for a 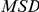 run. The second assumption is that the factor is not breaking down during the experimental period. The third one is the hypothesis of exponential growth for the number of cells 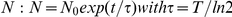. This assumption is well verified experimentally with 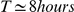 even for cells in the experimental sample dish. When the medium is exchanged by a slow dilution with a flow rate 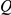, the following differential equation describes 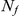:
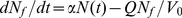
(2)


**Figure 4:**
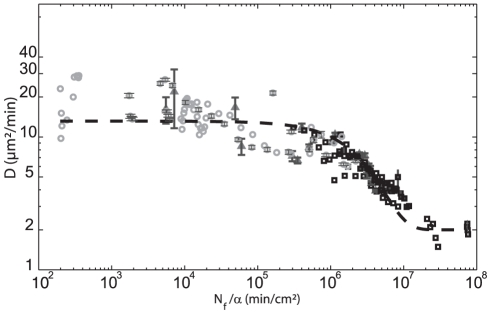
QSF density triggers cell motility: Summary of all experimental results. Diffusion Coefficient 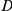 is plotted as a function of calculated 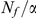 (with 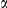, the rate of QSF secretion) for all experimental points: ‘aging’ experiments (square), cell density experiments (triangle), flow experiments (circle). All data are well fitted by the following equation 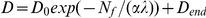; with 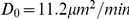, 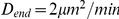, 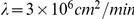, 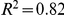. This empiric fit allows to easily define two plateaus at low and high density of QSF, respectively. For clarity, we reported only error bars on 3 representative experiments. Error bars represent SEM.

The first term is due to secretion, the second is due to dilution, 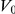 is the sample volume. When the sample medium is unchanged (*i.e.*, 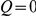), the solution 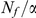 is exponential (aging effect, see supplementary material). Otherwise, the solution is a sum of two exponentials, the first is decreasing with time (*i.e.*, 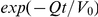) due to the dilution effect, the second one increasing with time (*i.e.*, 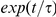) due to the aging effect, see supplementary material for the exact relations). These two exponentials explain why 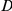 is first decreasing at low 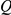 (aging is dominant) and increasing at high 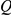 (dilution is dominant, Fig. 3D). With these equations it is possible to represent all 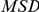 measurements as a function of the same common variable 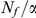 which spans over more than 6 decades (Fig. 4). The agreement between very different types of experiments (variable initial cell density 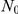, aging or experiment with a dilution rate) is very satisfactory. Interestingly the diffusion constant 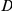 shows two plateaus values: a maximum value of about 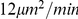 occurs at low QSF concentrations (very diluted cell density or dilution at high flows) while a minimum plateau value at 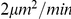 occurs at large QSF concentrations (HCM or aging experiments at very long times).

## Discussion

### Detection of Quorum Sensing Factor

We have shown in this study that a number of QSF directly governs cell migration. Thanks to a simple model based on rate of secretion and dilution (due to flow), all data could be reasonably fitted by a single master curve (Fig. 4). We mention again that data were obtained from a large set of independent experimental conditions (aging, constant and variable flow, stagnation, and conditioned media). Even with a very large variability in the individuals migrating properties, it is remarkable to see that the average migration rate is clearly governed by this QSF. The role played by such a factor in cell motility in the vegetative stage has never been reported to our knowledge. Even though we are unable to determine the absolute concentration of QSF in our experimental conditions, we can get information on cell sensitivity by analyzing the master curve. It is accurately fitted with an exponential decay with constant. For low QSF concentration, cells move with a constant high rate (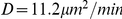) up to 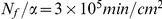. At this point, there is a sharp transition towards a lower rate of motion (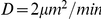), when 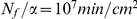. Using EqS.(5) (see supplementary material), we can estimate the corresponding cell density range for cells plated 2 hours before in fresh medium: from 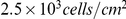 to 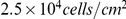 corresponding to a mean cell-cell distance of 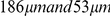, respectively. It is interesting to compare the lower critical cell density (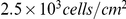) to the number of cells required for aggregation. It has been reported that a critical cell-cell distance of less than 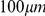 (*i.e.*, 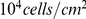) is necessary for cells to relay signals during chemotaxis and form aggregation streams. Increasing the distance between cells hinders their capacity to sense each other and relay cAMP signals [33]. Cells appear to have at least similar if not higher sensitivity to QSF than to cAMP. When cells are reaching the upper critical cell density (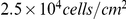), there is no further modification on cell motion, that stays low. This could be related to a saturation of receptors occupancy (equivalent to the one found during chemotaxis, where cell motility is depressed above a concentration in cAMP of 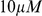, corresponding approximately to 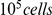 [34]).

It has to be pointed out that local cell density has a small influence on cell migration speed compared to the global concentration of QSF factor (Supplementary Fig. S6) and that we could not detect any self-organization of cells in territories (Supplementary Fig. S7). This is similar to chemotaxis, where signals relayed during aggregation (and hence locally modified with cell density) do not regulate individual cell speed [33]. We do not know at this stage the exact nature of this QSF. We cannot exclude that highly conditioned medium may become depleted of some nutrients (*e.g.* ATP, amino acids) or that the level of lactic acid may increase, even if cell proliferation seems not affected. Biochemical and molecular analysis of this QSF is beyond the scope of this paper. However, it should be interesting in future studies to analyze if this effect is related to the quorum-sensing mechanisms regulating adhesion found by Cornillon et al. [27]. Indeed, adhesion and migration are closely related in mammalian cells [35], and presumably also in *Dictyostelium* [36]. Undergoing experiments are analyzing the effect of QSF on adhesion. The two cell density sensing proteins identified during cell growth AprA and CfaD [26] could also be responsible for the regulation of cell motility described in this paper. Another possibility might be the cAMP itself, as it has been shown that some cAMP receptors are expressed at very low level in vegetative cells [37].

### Established and Novel Aspects of *Dictyostelium* Dynamics

It is difficult to compare quantitatively the numerous studies on *Dictyostelium* cell motion as experimental conditions (substrate, cell density, strain, buffer composition) were different. A general trend however is that amoeboid cells exhibit a correlated random walk: the direction of a cell's current movement is correlated with that of its movement in the past, and cells therefore move with persistence [14], [28], [38]. Van Haastert and Bosgraaf have extensively studied the ordered extension of pseudopods and found that they are of two types [11]: *(i)* splitting pseudopods extended at small angles and preferentially alternating to the right and left, causing the cell to take a persistent zigzag trajectory; *(ii)* de novo pseudopods extended in a random direction. The ratio of splitting pseudopods to de novo pseudopods determines the persistence of cell movement. Starved cells are faster than vegetative cells not by extending more pseudopods and/or increasing their speed but by moving longer in the same direction [39]. At small times, cells exhibit a complex dynamics with no simple exponential decay of the velocity autocorrelation and non Gaussian velocity distribution [7], [9], [10], [14]. At large times, in the case of aggregation competent cells, cells might be attracted by some nascent aggregation centers eventually not visible outside the recorded field of view. In the absence of external signals, 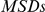 become satisfactorily fitted by the simple Fürth's formula (Eq.(1)) at times much larger than the persistent time [7]. If the experiments do not last long enough to self-average properly and to detect the long term features of highly persistent cells, it is difficult to distinguish true superdiffusion from correlated random walk [14], [40].

Due to the low magnification choice, it was not possible to observe accurately pseudopod dynamics, however we confirm for true vegetative cells in growth medium (HL5) the former conclusions on cell centroid motion: cells exhibit a correlated random walk with a persistence time of a few minutes. To fully observe this long term behavior, it is necessary to fit 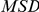 up to about 15 min and to track cells during at least three-fold this period (Supplementary Fig. S8). ‘Fast’ cells (with large 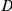) are more persistent than ‘slow’ cells. However, at short times we have found an interesting non-monotonous behavior in the velocity autocorrelation, with a negative peak at 2 min. This peak which has never been reported in the literature seems related to a tendency for many vegetative cells to retract initially extended pseudopods. Notice that it could be related to the oscillatory component of the velocity detected in Li *et al.* [14] although this oscillatory component was superimposed to a larger amplitude time average component of the velocity.

### Conclusion

This study clearly emphasizes that vegetative *Dictyostelium* cells display a classical persistent random motion at long times (*i.e.*, cells are persistent until a cross-over where they recover random diffusion). Extreme care has to be taken to analyze 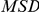 curves. Both the total recording time and the number of analyzed cells have to be large enough in order to obtain reliable statistics and reliable estimates of diffusion constant and persistent time. In agreement with numerous studies of *Dictyostelium* cells in a buffer, the diffusion constant appears strongly (positively) correlated with the persistent time indicating that cells modulate their migrated distance possibly by the proportion of persistent runs and not by their intrinsic speed. We demonstrate for the first time in the vegetative state that these quantities are regulated by a quorum sensing factor (QSF) secreted by cells. The molecular structure and the physiological role of this QSF remain to be elucidated but the existence of such a unique relation between cell motility and a QSF concentration and the reported methodology to obtain it (automated multisite cell tracking) offer opportunities to compare quantitatively various mutants and various environmental conditions (surface adhesion, stiffness of the substrate, medium composition).

## Materials and Methods

### Strain and Culture

*Dictyostelium Discoideum* DH1 were grown in HL5 medium (formedium) in plastic culture dishes (Falcon) at 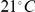. HL5 contains in g per liter: Peptone 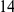; Yeast Extract 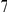; Glucose 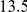; KH2PO4 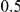; Na2HPO4 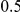. Vegetative cells were harvested at a density of 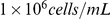, suspended in fresh medium and plated on glass coverslips at various densities (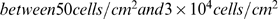) at 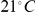.

### Microscopy

Glass coverslips were rinsed with 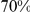 ethanol and HL5 medium. Custom-made wells were sealed on a glass coverslip allowing four different assays in parallel (each of area 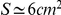, volume 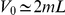). Renewing the cell medium was possible using a syringe pump (PHD ULTRA infuse/withdraw, Harvard Apparatus) connected to one or two wells. Cells were observed with an inverted light microscope (Nikon TE2000) using 4x objective and a phase contrast. A motorized x-y stage enabled to record in parallel up to 20 regions (*e.g.*, 5 different regions in each well). Images were captured at 30 seconds intervals for 8 to 12 hours.

### Image Processing

Recorded movies were binarized (see supplementary Fig. S1 and supplementary Movie S1) using ImageJ software with a custom made macro. Binarized movies were analyzed using Matlab to obtain individual cells positions (mass centroïd position), size and orientation.

### Motility Assay

Aging experiments: cells were placed on glass coverslips in a well filled with 2 mL of nutrient medium. After 1 hour letting cells settle on glass, they were tracked during 10 hours at 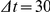 seconds intervals. The resulting 1200 images were organized in stacks of 100 images; 50 minutes is the lowest time interval to get a reasonable diffusion coefficient. Other ‘Aging’ experiments were performed using highly conditioned medium (from cell cultured during 24 hours) or moderately conditioned medium (from cell cultured at experimental density for 4 hours). For flow experiments, cell medium was renewed using either an exponential flow ramp renewing medium from 0 up to 0.5 mL/min either a constant flow rate renewing up to 2 mL/min.

### Measurement of Motion Parameters

For a set of 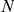 cells tracked during 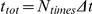, the mean-squared displacement (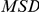) is calculated as a function of the time lag 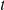:
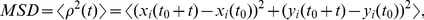
(3)where we take the average over 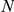 and over all possible 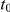 using overlapping intervals [41]. We also calculated standard error (SD) and standard error of the mean (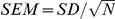). 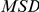 were fitted with Eq.(1) for time larger than 5 min. Error on the fitted diffusion constant 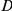 were estimated from the SEM of the 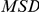 at 15 min. The comparison of the diffusion coefficients obtained between different cell density ranges of Fig. 3A was performed with a Wilcoxon non parametric test. P values 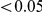 were considered significant (* indicating 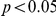, ** indicating 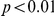). We used the instantaneous velocity of the cell 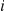 at time 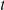, 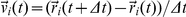 to calculate the normalized or non-normalized autocorrelation function of the velocity 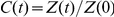 and 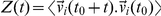, respectively. Bimodal analysis was performed as described in [42]. Briefly, the turn angle 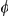 was defined as the angle between 2 successive centroïd vector displacements. If, at least, 3 successive 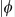 are below a threshold set as 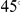, this portion of the trajectory is considered as persistent. Otherwise, it is random (see Supplementary Fig. S4). For each cell, we computed the proportion of time spent in persistent mode 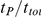, the mean distance of each persistent track 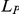 and the total distance traveled in persistent mode 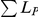. Error bars on these quantities represents SEM.

## Supporting Information

Figure S1
**Cell contour detection.** Detail (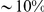) of one field of view obtained by bright field microscopy using a 4x objective lens (**A**) which is superimposed with cell contours identified by our ImageJ script (**B**). In (**C**) the corresponding binary image is displayed.(EPS)

Figure S2
**Cell shape is correlated with migrated distance.** (**A**) time lapse of 2 cells (upper and lower rows:slow and fast, respectively). Despite the low resolution, we can distinguish easily that the fast cell is more elongated and more active than the slow cell. Cell roundness (**B**) and roundness difference (**C**) between two successive images are correlated with the diffusion constant (positively and negatively respectively). Squares correspond to aging experiments, circles to experiments with a flow. The two colored boxes highlight two special groups of cells, very slow and fast respectively. Roundness is defined as 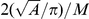 where 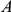 is the cell area and 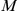 the cell major axis length (both are determined with Matlab). Each point corresponds to the average over all cells tracked during a single experiment. The more elongated the cells are (Roundness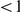) the faster they move. Roundness difference quantify the cell deformation activity. The larger is this activity, the faster move the cells.(EPS)

Figure S3
**Noise on positions.** (**A**) 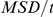 *Vs.* 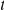 at 4x (blue squares) and 20x (green squares) magnification calculated for cells. 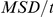 of fixed dusts are represented by red and green dots for 4x and 20x respectively. Black diamonds represent 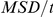 calculated from cells trajectories corrected with the drift of dust coordinates. Dotted lines are fits using the Fürth's formula (Eq.1 in the main manuscript) of 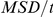. The fit is calculated from data points in the range [500–1000] sec but is represented in the full range [0–1000] sec to appreciate the overshoot height. (**B**) Velocity autocorrelation *vs.* time for cells at 4x (blue squares) and 20x (green squares) magnification, and dust at 4x magnification (red line). The inset gives a larger view of the negative peaks. Concerning dusts, the negative peak due to camera and repositioning errors occurs around 30 seconds (the time interval used to record dusts movement). Cells speed autocorrelation shows a negative peak around 2 min which cannot be explained by position errors.(EPS)

Figure S4
**Cell tracking and Bimodal analysis.** Typical tracks of persistent mode (in red) and random mode (in black) along a cell trajectory lasting 50 min (time step between two points, 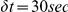), starting point (green square).(EPS)

Figure S5
**Individual cell distributions of the persistence parameters obtained from a bimodal analysis.** 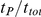 is the proportion of time spent in persistent mode, 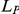 is the mean persistent run length and 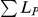 the cumulated distance in the persistent mode. Three different experiments corresponding to the ones of Fig. 1D are represented: in red (**A**–**C**), histograms of fast moving cells; in blue (**D**–**F**),histograms of cell moving at intermediate speed; in gray (**G**–**I**), histograms of slow moving cells. The solid line is a fit of each histogram with a Gaussian function. All fits of a given parameter are superimposed in (**G**–**I**) to better visualize the differences between the three experiments. Histogram reveals large distribution of cells within an experiment but clear differences between mean values of each parameters, reinforcing the use of large statistics to describe cell motility. The faster the cells, the larger are the persistence parameters.(EPS)

Figure S6
**Effect of the local density.** Local density has little effect if any on the effective diffusion constant defined from t = 15 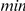 as 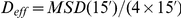. Data originate from different regions of the same sample dish with different local cell densities. Red bullets correspond to the region of low local density (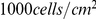), black bullets to a high local density (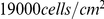) and green bullets correspond to a region with an intermediate density probably more representative of the mean density within the well (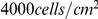). Cells with a local low density move slightly faster than cells with a local higher density at least 2 h after cells were transferred to fresh medium. The difference however is small as compared to the changes with time due to the aging effect.(EPS)

Figure S7
**Pair correlation function analysis rules out any cell-cell structuration.** Three typical experiments at the same time after cells were plated (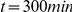) and at the same cell density (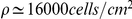) but with different migration rates due to different media are presented. Black crosses correspond to very slow cells (highly conditioned medium, 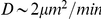), blue circles correspond to cells moving at intermediate speed (aging experiment, 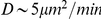) and red squares to fast cells (flow experiment, 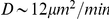). The pair correlation function is defined as 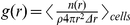 where 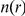
is the mean number of cells in a ring of width 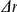 at distance 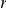. Fast cells are randomly organized (flat landscape). Slower cells present a peak at 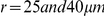 for cells in HCM or HL5 medium respectively. These positions are significantly smaller than the mean cell-cell distance obtained from the mean density 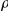. This structuration is probably due to a lack of diffusion of cells after cell division (*i.e.*, clustering effect) not a real self-organization.(EPS)

Figure S8
**Error due to number of cell ***N***, total time t_tot_ and time interval ***Δt***.** (**A**) RSE of 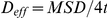 at 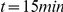 *Vs.* 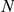 calculated by generating and averaging 1000 ramdom samples of 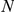 among the full recorded cells. Inset: Standard deviation of 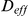 *Vs.* 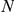 (same procedure). The black line represent the average of 1000 random samples and diamonds a single random sample. (**B**) Standard deviation *Vs.* 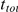 for 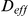 calculated at 5 min (blue), 15 min (black) and 30 min (red). (**C**) Standard Deviation of 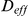 calculated at 15 min as a function of time interval 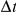.(EPS)

Movie S1 Movie S1 represents binarization for typical *Dictyostelium* cells at 4x magnification in nutrient medium.
(AVI)

Movie S2 Movie S2 shows fast and slow cells migration in parallel. Cells are at the same density for both conditions. This movie is representative of the Trajectories shown in Fig. 1B–C.
(AVI)
